# Multimodal Assessment of Vascular and Ventricular Function in Children and Adults With Bicuspid Aortic Valve Disease

**DOI:** 10.3389/fcvm.2021.643900

**Published:** 2021-03-23

**Authors:** Constance G. Weismann, Sara Ljungberg, Anna Åkesson, J Hlebowicz

**Affiliations:** ^1^Division of Pediatric Cardiology, Clinical Sciences, Skåne University Hospital, Lund University, Lund, Sweden; ^2^Clinical Studies Sweden – Forum South, Skåne University Hospital, Lund, Sweden; ^3^Department of Cardiology, Clinical Sciences, Skåne University Hospital, Lund University, Lund, Sweden

**Keywords:** bicuspid aortic valve, aortic stiffness and distensibility, augmentation index, pulse wave velocity, intima media thickness, endothelial function, diastolic function, congenital heart disease

## Abstract

**Background:** Bicuspid aortic valve (BAV), the most common congenital cardiac anomaly, has been associated with an aortopathy, increased aortic stiffness and diastolic dysfunction. The involved mechanisms and impact of age remain unclear. It was the aim of this study to characterize arterial and cardiac function, their correlation, and the effect of age in children and adults with a history of BAV.

**Methods:** Multimodal cardiovascular assessment included echocardiography, ascending aortic distensibility, common carotid intima media thickness [cIMT], parameters of wave reflection [central (cAIx75) and peripheral (pAIx75) augmentation index corrected to a heart rate of 75/min, aging index (AI)], carotid-femoral pulse wave velocity [cfPWV], and endothelial function (EndoPAT). Multivariable linear regression and correlation analyses were performed.

**Results:** We included 47 BAV patients and 84 controls (age 8–65 years). Ascending aortic stiffness, pulse wave reflection (cAIx75, pAIx75, and AI) and central blood pressure were significantly increased in patients with BAV. However, PWV, cIMT, and endothelial function were not significantly different from controls. BAV patients had marginally reduced diastolic (E': β = −1.5, *p* < 0.001) but not systolic function compared to controls. Overall, all parameters of arterial stiffness had moderate-strong correlations with diastolic dysfunction and age. In the BAV group, ascending aortic distensibility had the strongest correlation with diastolic dysfunction.

**Conclusions:** BAV is associated with increased proximal arterial stiffness and wave reflection. However, PWV and cIMT are not increased, and endothelial function is preserved. This suggests that the mechanism of arterial and cardiac stiffening is different from patients with acquired heart diseases.

## Introduction

Bicuspid aortic valve (BAV) is one of the most common congenital cardiac malformations, existing in 1–2% of the population. Due to its high prevalence, it may cause more morbidity and mortality than all other congenital heart defects combined (1). BAV has a wide spectrum of clinical presentations ranging from the neonate with critical aortic stenosis to the asymptomatic adult. The disease is, however, not confined to the aortic valve, but rather associated with a congenital aortopathy which is thought to predispose to ascending aortic stiffening, dilation and even dissection (2–4). Furthermore, diastolic and systolic dysfunction have been described in BAV patients even without significant valvular impairment (3, 5, 6). The cause for diastolic and/or systolic myocardial dysfunction without significant valve dysfunction remains unclear, but could be due to (1) abnormal intraventricular flow dynamics in the setting of an asymmetrically opening valve, (2) intrinsic myocardial abnormalities, or (3) be the result of arterio-ventricular interaction (i.e., the impact of arterial stiffening on ventricular function). Arterio-ventricular interaction as a cause of diastolic heart failure appears to be an important mechanism in adults with heart failure and preserved ejection fraction (7). There, arterial stiffness increases late systolic afterload, which in turn affects thick-thin myofilament interaction and crossbridge dissociation, leading to impaired cardiac relaxation during diastole (7). However, the published data on arterio-ventricular interaction specific to congenital heart disease and BAV in particular are limited and controversial to date (3, 6, 8).

We hypothesized that patients with BAV have abnormalities in vascular characteristics that extend from the large to small arteries, and that aortic stiffness correlates with left ventricular diastolic function. The secondary hypothesis was that arterial and ventricular stiffening are present in childhood and are further increased at older age. We tested these hypotheses using a multimodal approach on children and adults with a history of BAV, aiming to enhance insights into the pathophysiological mechanisms involved in BAV disease. The methods chosen aimed to cover anatomical and physiological aspects ranging from the ascending aorta to peripheral arteries.

## Methods

### Study Population

This is a prospective cross-sectional observational study comparing cardiovascular function in patients with a history of a BAV to healthy controls. The study was approved by the local ethics committee (#2017/243) and conducted 2017-2019.

BAV patients were recruited through SWEDCON (Swedish national registry on congenital heart disease) and control patients were recruited through advertisement. Inclusion criteria for the BAV group were a history of BAV including patients who have undergone aortic surgery such as commissurotomy, aortic valve or ascending aortic replacement. Exclusion criteria were associated congenital heart disease (e.g., aortic coarctation), severe aortic stenosis or insufficiency, cardiac surgery within the last 3 months, diabetes, rheumatological, hematological, or oncological disorders. Control group specific exclusion criteria were a personal history of heart disease or a family history of a 1st degree relative with known thoracic aortic aneurysm.

Baseline characteristics such as gender, age, weight, height, body surface area, body mass index, blood pressure and heart rate were recorded. For the BAV group, prior cardiac interventions were documented if applicable. Patients were examined after at least 4 h of fasting and a minimum 10 h abstinence from caffeine and nicotine. The exams were performed in a quiet room with dampened light at a room temperature set to 22°C. Patients were in a supine position for at least 5 min prior to vascular examinations.

### Ultrasound

Echocardiograms and common carotid artery ultrasound evaluations were performed using the EPIQ7 (Philips Healthcare, Netherlands). Probe frequency (X5-1, X7-2, L15-7io) was selected as appropriate for patient size. Echocardiograms were performed using 2-dimensional, color, spectral, and tissue Doppler (TDI) as previously described (9). For TDI measurements, averages of septal and lateral E' velocities [cm/s] were used. Four-dimensional analysis of left ventricular systolic function included ejection fraction (EF). Four beat acquisitions were obtained at a frame rate of at least 30 Hz.

As previously described, two-dimensional measurements of the ascending aorta in peak systole (SD) and end-diastole (DD) were obtained from a high parasternal long axis view at the level of the right pulmonary artery to calculate distensibility, stiffness index, and strain (9, 10). Patients who had undergone prior ascending aortic replacement were excluded for ascending aortic elasticity measurements. Mean common carotid intima media thickness (cIMT) was measured semi-automatically in end-diastole over a distance of 1 cm, using a 15 MHz transducer.

Measurements were performed offline (Philips Intellispace and QLAB Cardiac Analysis, Philips Healthcare, Netherlands; 4D LV-Analysis, Tomtec Imaging Systems, Unterschleissheim, Germany). All measurements were performed by one of two experienced congenital echocardiographers (B.G., C.W.).

### Arterial Function

Carotid-femoral arterial pulse wave velocity (PWV), a surrogate parameter of large arterial stiffness, was determined using SphycmoCor XCEL (AtCor, Australia) (11). Path length was measured according to guidelines, using the direct method ^*^ 0.8 (12). CfPWV was recorded over a period of 10 s. Averages of two separate measurements were used for analyses. Only measurements that passed the internal Quality Control were used.

Using the same device, pulse wave analysis was performed, which uses a transfer function to derive a central from a brachial pulse wave form. Cuff size was selected according to arm circumference. Using the central wave form, central blood pressure and augmentation index (cAIx) corrected to a heart rate of 75 beats per minute (cAIx75) were determined. AIx is defined as the difference between the reflected wave (P2) and the forward wave (P1), divided by the pulse pressure. A higher cAIx75 corresponds to a relatively increased wave reflection and stiffer arteries. Following two consecutive right arm blood pressure measurements, pulse wave analysis was performed and averaged over 10 s. The protocol was repeated and average measurements were used for analyses.

Digital pulse wave analysis with photoplethysmography (DPA; Meridian, South Korea) provides a digital pulse curve as well as its second derivative that represents accelerations and decelerations of the blood flow (Acceleration Plethysmography) (13). Aging index (AI) is derived from the acceleration curve and has previously been shown to correlate strongly with AIx75 measured by the SphygmoCor device (13). A higher, less negative AI is consistent with aging (i.e., stiffer arteries). The second derivative of the pulse curve in the right index finger was analyzed continuously over 1 min. Averages of two separate measurements were used for analyses.

Endothelial function was assessed using EndoPAT 2000 (Itamar Medical, Israel) (14, 15). The reactive hyperaemic response in the right index finger was measured following a 5 min period of right arm occlusion in relation to baseline and contralateral peripheral arterial tone (reactive hyperemia index, RHI) (16). From the baseline recording, the peripheral augmentation index [(P2-P1)/P1] corrected to a heart rate of 75 beats per minute (pAIx75) was derived.

### Statistics

For statistical analyses, continuous variables were expressed as median and inter-quartile range (IQR). Categorical variables were expressed as frequency and compared by the Chi square or Fisher exact test as appropriate. Linear regression analyses were carried out correcting for the covariates age and sex. Additional covariates were included in the model as appropriate and specified in **Tables 2**–**4**. If necessary, logarithmic transformation was used (stiffness index). Variables were associated using Pearson's correlation coefficient (*r*). A *p*-value of <0.05 was considered statistically significant. Where appropriate, Bonferroni correction of alpha level was used to adjust for multiple comparisons. Data were stored using REDCap electronic data capture tools hosted at Lund University. Statistical analysis was performed using Statistical Package for Social Sciences, version 25 (IBM SPSS, Chicago, IL).

## Results

We prospectively recruited 47 patients with a history of BAV and 88 controls. Of the controls, three were excluded due to pre-existing cardiovascular disease, and one was excluded due of technical difficulties. Thus, 84 controls were included in the study. The median age was 29 (range 8–65) years. Eleven patients were under 18 years of age.

### BAV Cohort Description

The majority of the patients had never required an intervention for BAV. Twenty (43.6%) of the 47 subjects had at least one intervention. Eight (17%) had undergone surgical commissurotomy. Fifteen (32.0%) had prosthetic aortic valves (five mechanical, two bioprosthetic, five Ross procedures) whereof six (12.8%) also had ascending aortic grafts. No one had been operated for aortic dissection. At the time of the study visit, four (8.5%) patients had moderate aortic stenosis with mean gradients between 20 and 30 mmHg, and two (4.3%) had moderate aortic insufficiency associated with BAV. Only 12 (25.5%) of the patients with BAV were taking medications (anticoagulants: *n* = 7, 14.9%; antihypertensives: *n* = 3, 6.4%).

### Demographics BAV vs. Control

There was no difference between the BAV group and the control group regarding age, weight, height, BMI, HR and nicotine use. However, the BAV group had significantly higher proportion of males as well as higher systolic and diastolic brachial blood pressures (Table 1). Following correction for age and sex though, systolic blood pressure no longer significantly elevated (*p* = 0.233), while diastolic blood pressure was significantly elevated (β = 3.4, *p* = 0.032).

**Table 1:** Comparison of demographic characteristics between BAV patients and controls.

**Demographic characteristics**	**BAV (*n* = 47)**	**Controls (*n* = 84)**	***p***
Age [years]	36 (19–46)	27 (20–40)	0.125
Female gender	14 (30 %)	42 (50%)	0.025
Height [cm]	176 (170–182)	171 (160–180)	0.079
Weight [kg]	74 (62–86)	70 (58–80)	0.139
Body mass index [kg/m^2^]	23 (21–25)	23 (21–27)	0.623
Heart rate [beats per minute]	61 (56–69)	60 (54–68)	0.308
Brachial systolic blood pressure [mmHg]	123 (116–135)	118 (112–123)	0.013
Brachial diastolic blood pressure [mmHg]	77 (69–84)	70 (64–77)	0.003
Nicotine use	9 (19%)	12(15%)	0.506

*Values expressed as median (interquartile range). Significance was tested with Mann-Whitney U-test. P-value of <0.05 was considered significant*.

### Cardiac Function

Left ventricular mass index was significantly greater and diastolic function was significantly impaired compared to healthy controls following adjustment for age and sex (Table 2). This was evidenced by a lower E' velocity by TDI and a higher E/E' ratio. The difference in diastolic function (E') remained statistically significant when adding moderate aortic stenosis (*b* = −1.2, *p* = 0.001), LV mass index (*b* = −1.4, *p* < 0.001), or central blood pressure (*b* = −1.4, *p* < 0.001), as covariates. In the BAV group, diastolic function correlated negatively with LV mass index (*r* = −0.36, *p* = 0.015), but not with left ventricular outflow tract gradient (*p* = 0.788). In addition, E' corrected for ascending aortic distensibility was not significantly different between the BAV and control groups (*p* = 0.399). Systolic function described by 4-dimensional EF was not significantly different between the groups. Above findings on cardiac structure and function were sustained when excluding BAV patients who have undergone prior aortic valve replacement (Table 3).

**Table 2:** Descriptive statistics and linear regression model comparing outcome variables of BAV patients to controls.

	**BAV (*n* = 47)**	**Controls (*n* = 84)**	**β (95% CI)**	***p***
**Diastolic and systolic cardiac function**				
E' [cm/s]	11.8 (9.6–13.1)	13.6 (11.8–15.1)	−1.5 (−2.2 to −0.8)	<0.001
E/E' ratio	7.6 (6.3–9.2)	5.8 (5.1–6.7)	2.0 (1.4 – 2.7)	<0.001
Ejection fraction [%]	60.9 (55.9–63.7)	62.3 (59.9–64.5)	−1.5 (−3.2 – 0.2)	0.084
Left ventricular mass Index [g/m^2^]	83.2 (61.7–98.9)	65.96 (49.4–75.4)	19.1 (11.0 – 27.3)	<0.001
**Proximal arterial characteristics by ultrasound**				
Ascending aortic dimension [cm]	3.4 (2.9–4.1)	2.9 (2.5–3.1)	0.7 (0.5 – 0.9)	<0.001
AscAo distensibility [10^−6^cm^2^/dyn]	2.2 (1.6–3.4)	5.2 (4.2–7.1)	−2.3 (−3.0 to −1.6)	<0.001
AscAo stiffness index	9.0 (6.4–14.4)	4.1 (3.1–5.4)	2.7 (2.5 – 2.9)	<0.001
AscAo strain [%]	5.6 (3.6–7.8)	12.3 (9.2–17.2)	−5.9 (−7.7 to −4.2)	<0.001
cIMT [mm]	0.5 (0.4–0.6)	0.5 (0.4–0.6)		0.939
**Central blood pressure, arterial, endothelial and microcirculatory function**				
Central systolic pressure [mmHg]	110.5 (103.5–120.3)	102.5 (95.8–109.0)	5.6 (1.8–9.4)	0.004
Central diastolic pressure [mmHg]	77.5 (70.0–82.3)	70.8 (65.0–77.1)	3.7 (0.6–6.8)	0.020
cAIx75%^§^	10.1 (−1.3–23.3)	−6.3 (−14.47–6.82)	15.1 (9.9–20.1)	<0.001
pAIx75%^§^	−1.00 (−13.00–15.00)	−18.00 (−27.00 to −3.75)	13.8 (8–19.5)	<0.001
Aging index^*§^	−0.50 (−0.73 to −0.13)	−0.73 (−0.94 to −0.58)	0.2 (0.1–0.3)	<0.001
Pulse wave velocity [m/s]^*§^	6.2 (5.1–7.6)	6.6 (4.9–7.2)		0.182
Reactive hyperemia index	2.3 (1.8–2.6)	2.2 (1.7–2.6)		0.748

Ascending aortic parameters excluded subjects who have undergone ascending aortic replacement. Continuous variables are presented as median (interquartile range), linear regression results as β (95% confidence interval). Beta values adjusted for age and sex, and their significance level (p) are provided if p < 0.1. Parameter specific additional covariates are marked as: central mean pressure, ^§^height, ^*^heart rate.

*AscAo, ascending aorta; cIMT, common carotid artery intima media thickness; cAIx75, central augmentation index corrected to a heart rate of 75/min; pAIx75, peripheral augmentation index corrected to a heart rate of 75/min*.

**Table 3:** Subgroup analysis of BAV patients with native aortic valves (i.e., excluding 15 patients who have undergone prior aortic valve replacements) compared to controls.

	**β**	***P***
**Diastolic and systolic cardiac function**		
E' [cm/s]	−1.4	<0.001
E/E' ratio	1.7	<0.001
Ejection fraction [%]		0.572
LV mass index [g/m^2^]	12.6	0.003
**Central blood pressure and proximal arterial characteristics by ultrasound**		
Ascending aortic dimension [cm]	0.7	<0.001
AscAo distensibility	−2.4	<0.001
AscAo stiffness index	2.9	<0.001
AscAo strain [%]	−5.8	<0.001
CCA IMT [mm]		0.133
**Arterial, endothelial and microcirculatory function**		
Central systolic pressure [mmHg]	5.2	0.008
Central diastolic pressure [mmHg]	3.7	0.033
cAIx75%^§^	17.0	<0.001
pAIx75%^§^	15.4	<0.001
Aging index^*§^	0.3	<0.001
Pulse wave velocity [m/s] ^*§^		0.292
Reactive hyperemia index		0.928

Linear regression model adjusting for age, sex, and parameter specific additional variables marked as: central mean pressure, ^§^height, *heart rate. β is provided if p < 0.1.

### Arterial Characteristics

Multimodal assessment of arterial function consistently revealed pathologic changes of proximal arterial characteristics and wave reflection in the BAV group (Table 2). The proximal large arteries were characterized by decreased ascending aortic elasticity (increased stiffness index, decreased distensibility, and strain). CIMT, by contrast, was not significantly different from controls. Central blood pressure and arterial wave reflection measured by three different methods (cAIx75 by SphygmoCor XCEL, pAIx75 by EndoPAT, and AI by DPA) was significantly increased in BAV patients compared to controls. In spite of clearly increased proximal arterial stiffness and increased peripheral wave reflection, cfPWV – representing arterial stiffness between the carotid and femoral arteries—was not significantly different between patients and controls (Table 2). Further, RHI, which describes endothelial function, revealed no difference between the BAV and Control groups. Above findings on arterial characteristics were sustained when excluding BAV patients who have undergone prior aortic valve replacement (Table 3).

### Arterial and Cardiac Stiffening Independent of Prior Intervention

In order to identify risk factors for worse diastolic function (average E'), ascending aortic distensibility or cAIx75we performed multivariate linear regression analyses within the BAV group. Independent variables included age, sex, central systolic pressure, LV mass index, aortic valve morphology, moderate aortic stenosis, history of prior aortic valve intervention, aortic valve prosthesis, and prior ascending aortic replacement. Except for age (see above) none of the other factors met statistical significance (data not shown).

In an effort to evaluate potential differences in diastolic or arterial function that are secondary to prior surgery, we performed subgroup analyses comparing BAV patients with at most mild valve dysfunction and no prior aortic procedure (BAV_1; *n* = 22) to BAV patients who have had an aortic procedure (BAV_2; *n* = 19) to controls. Following Bonferroni correction for multiple comparisons, differences in diastolic function (average E') were no longer significant between the BAV subgroups and controls (Table 4). By contrast, group differences in ascending aortic distensibility (in those with native ascending aortas) and cAIx75 prevailed, but there was no significant difference in arterial parameters between the BAV subgroups (Table 4).

**Table 4:** Subgroup analysis comparing BAV patients with native well-functioning valves (BAV_1; *n* = 22) and BAV with prior intervention (BAV_2; *n* = 19) to controls.

	**BAV_1 vs. Control**		**BAV_2 vs. Control**		**BAV1_vs. BAV_2**	
	**β**	***P***	**β**	***p***	**β**	***p***
E' [cm/s]	−1	0.096	−1.3	0.045		1
AscAo distensibility	−2.2	<0.001	−2.5	<0.001		1
cAIx75%^§^	13.5	<0.001	15.1	0.001		1

*Linear regression model with Bonferroni correction, adjusting for age and sex. Parameter specific additional variables marked as: ^§^height, β is provided if p < 0.1*.

### Arterio-Ventricular Interaction

**Figure 1:**
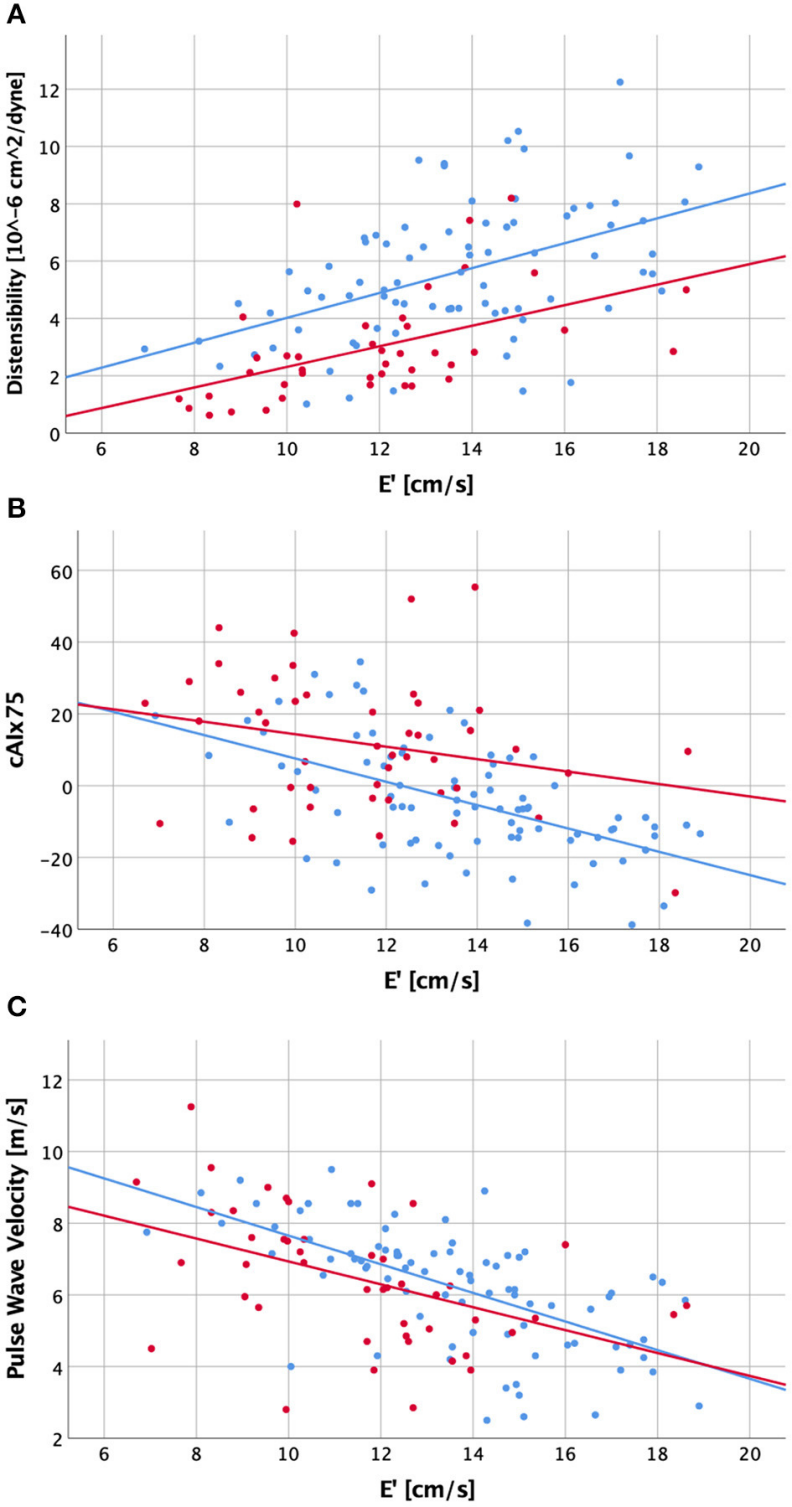
Scatter plot visualizing correlations between diastolic function (E') and ascending aortic distensibility **(A)**, central augmentation index corrected to a heart rate of 75/min [cAIx75; **B**] and carotid-femoral pulse wave velocity **(C)** for patients with a history of bicuspid aortic valve (red dots and regression line) and healthy controls (blue dots and regression line).

Next, we evaluated whether diastolic cardiac function correlates with arterial characteristics, correcting for the effect of aortic stenosis (Table 5, Figure 1). Across all study subjects, there were moderate-strong and highly significant correlations between diastolic function and absolute values of arterial parameters.

**Table 5:** Correlations between average E' (diastolic function) and cardiovascular characteristics for all patients as well as group-wise analysis for controls and BAV patients.

**Diastolic function**	*R* for All*	***p***	*R* for BAV*	***p***	***R* for Control**	***p***
	***N* = 131**		***N* = 47**		***N* = 84**	
**Proximal arterial characteristics by ultrasound**						
AscAo Distensibility [10^−6^cm^2^/dyn]^†^	0.59	<0.001*	0.63	<0.001*	0.48	<0.001*
Ascending aortic dimension [cm]^†^	−0.54	<0.001*	−0.37	0.016	−0.58	<0.001*
cIMT [mm]	−0.59	<0.001*	−0.53	<0.001*	−0.65	<0.001*
**Arterial wave reflection and pulse wave velocity**						
Central systolic blood pressure [mmHg]	−0.54	<0.001*	−0.34	0.031	−0.61	<0.001*
cAIx75%	−0.47	<0.001*	−0.23	0.149	−0.54	<0.001*
pAIx75%	−0.40	<0.001*	−0.17	0.301	−0.45	<0.001*
Aging Index	−0.42	<0.001*	−0.38	0.031	−0.40	<0.001*
Pulse Wave Velocity [m/s]	−0.56	<0.001*	−0.47	0.002*	−0.63	<0.001*

Pearson's correlation coefficient R and p-values are listed. *Significant following Bonferroni correction for multiple comparisons with significance level at 0.006.

*AscAo, ascending aorta; cIMT, common carotid artery intima media thickness; cAIx75, central augmentation index corrected to a heart rate of 75/min; pAIx75, peripheral augmentation index corrected to a heart rate of 75/min*.

six patients with aortic prostheses excluded. *Partial correlations adjusting for moderate aortic stenosis.

We then evaluated correlations between arterial parameters and diastolic function group-wise. In decreasing order, the control group had moderate strong and highly significant negative correlations of diastolic function with cIMT, PWV, central systolic pressure, ascending aortic dimension, cAIx75, inverse ascending aortic distensibility, pAIx75 and AI (|r| = 0.4–0.65). In the BAV group, by contrast, diastolic function had significant correlations (in decreasing order) only with ascending aortic distensibility, cIMT and PWV (|r| = 0.47–0.63), while central systolic pressure, ascending aortic dimension, cAIx75, pAIx75, and AI did not meet statistical significance following Bonferroni correction. Ascending aortic dimension corrected for distensibility, age and sex did not reveal significant correlations with diastolic function or cAIx75 in either group.

### Arterial and Cardiac Stiffening With Advancing Age

**Figure 2:**
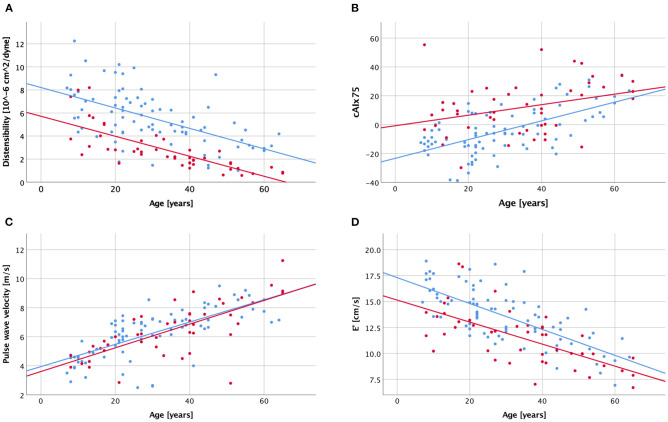
Scatter plot visualizing correlations between age and ascending aortic distensibility **(A)**, central augmentation index corrected to a heart rate of 75/min [cAIx75; **B**], carotid-femoral pulse wave velocity **(C)**, and diastolic function [E'; **D**] with a history of bicuspid aortic valve (red dots and regression line) and healthy controls (blue dots and regression line).

All arterial parameters tested had strong and highly significant correlations with age in the control group (Table 6, Figure 2). In the BAV group, however, inverse ascending aortic distensibility, cIMT, central systolic pressure, and PWV correlated strongly (*r* > 0.7, *p* < 0.001), and AI moderately with age. CAIx75, pAIx75 and ascending aortic dimension did not have significant correlations with age in the BAV group following Bonferroni correction for multiple comparisons.

**Table 6:** Correlations between age and cardiovascular characteristics for all patients as well as group-wise analysis for BAV patients and controls.

**AGE**	***R* for All**	***p***	***R* for BAV (Control)**	***p***	***R* for Control**	***p***
	***N* = 131**		***N* = 47**		***N* = 84**	
**Proximal arterial characteristics by ultrasound**						
AscAo Distensibility [10^−6^cm^2^/dyn]	−0.60	<0.001*	−0.75	<0.001*	−0.57	<0.001*
Ascending aortic dimension [cm]	0.45	<0.001*	0.33	0.038	0.66	<0.001*
cIMT [mm]	0.75	<0.001*	0.79	<0.001*	0.71	<0.001*
**Arterial wave reflection and pulse wave velocity**						
Central systolic blood pressure [mmHg]	0.60	<0.001*	0.61	<0.001*	0.58	<0.001*
cAIx75	0.51	<0.001*	0.33	0.025	0.62	<0.001*
pAIx75	0.48	<0.001*	0.34	0.024	0.55	<0.001*
Aging Index	0.52	<0.001*	0.47	0.001*	0.55	<0.001*
Pulse Wave Velocity [m/s]	0.71	<0.001*	0.72	<0.001*	0.70	<0.001*
**Cardiac function**						
E' [cm/s]	0.70	<0.001*	−0.66	<0.001*	−0.72	<0.001*
Ejection fraction [%]	−0.20	0.026	−0.36	0.015	−0.02	0.833

Pearson's correlation coefficient R and p-values are listed. ^*^Significant following Bonferroni correction for multiple comparisons with significance level at 0.005.

*AscAo, ascending aorta; cIMT, common carotid artery intima media thickness; cAIx75, central augmentation index corrected to a heart rate of 75/min; pAIx75, peripheral augmentation index corrected to a heart rate of 75/min*.

*six patients with aortic prostheses excluded*.

Diastolic function (average E') was decreasing with age in both groups, while a trend toward decreasing systolic function (EF) was seen only in the BAV group (Table 6, Figure 2D).

## Discussion

This multimodal study of children and adults with a history of BAV disease demonstrates increased proximal aortic stiffness and wave reflection while there is no evidence of generally increased arterial stiffness, or endothelial dysfunction. Ascending aortic distensibility and diastolic function correlate with each other, are reduced already at young age, and decline further with advanced age. Arterial wave reflection, by contrast, is abnormal already at young age, does not worsen significantly with advanced age and does not correlate significantly with diastolic function. Overall, diastolic function appears only marginally decreased—though statistically highly significant—compared to controls, and can likely be attributed to decreased ascending aortic distensibility.

Proximally increased aortic stiffness has been described previously in children and adults with BAV, suggesting that impaired aortic elasticity may be congenital (3, 4, 17). In addition, Lee et al. have previously shown increased AIx75 in BAV disease (18). Most recently, we demonstrated in patients with repaired aortic coarctation, that those with associated BAV har particularly elevated Aix75 (19). In the study presented herein, multimodal assessment of vascular function revealed that vascular impairment appears indeed limited to the proximal aorta. The underlying mechanism of increased proximal wave reflection (as evidenced by increased cAIx75, pAIx75, and AI) may be due to a combination of eccentric flow across the BAV, ascending aortic dilation and increased ascending aortic stiffness. This hypothesis is supported by a recent cardiac magnetic resonance imaging study where BAV was associated with higher viscous energy loss compared with healthy controls (20).

By contrast, arterial abnormalities that are usually seen with arteriosclerotic changes, were not detected in our BAV cohort. First, cfPWV was not increased compared to controls. Both, increased as well as normal cfPWV have previously been described in BAV with dilated compared to non-dilated aortas (18, 21). Secondly, cIMT was not increased in patients with BAV. Similarly, Goudot et al. recently found no altered carotid artery stiffness compared to healthy controls when measuring carotid distensibility, maximal rate of systolic distension, and local PWV (22). Interestingly—as in our cohort—these patients did have increased ascending aortic stiffness. This supports the notion that the stiffness of the aorta in BAV is not due to arteriosclerotic changes. Third, we found no evidence of endothelial dysfunction in the small arteries (EndoPAT). Endothelial dysfunction, assessed by flow mediated dilatation (FMD) in the brachial artery, however, has been described in patients with BAV (17, 21). Thus, endothelial function may be affected in the brachial artery but not in smaller vessels.

There has been conflicting data about whether or not aortic stiffness correlates with diastolic function in BAV patients (3, 6, 8, 18). The current study was unique in that we used multiple modalities to answer this question. While diastolic function in the control group correlated moderately-strongly with all arterial parameters, the only significant correlations in the BAV group—controlling for aortic stenosis—were seen with ascending aortic distensibility, cIMT and cfPWV. In absolute terms, diastolic impairment in the BAV group was at most modest following adjustment for aortic stenosis. In fact, E' corrected for ascending aortic distensibility was not significantly different between the BAV and control groups (*p* = 0.399), suggesting that changes in E' across BAV patients and controls can be attributed to changes in ascending aortic distensibility. These findings argue against an intrinsically increased myocardial stiffness.

Systolic function was overall preserved in patients with BAV, but there was a trend toward a lower EF with increasing age. In an earlier study, EF has been described to be lower in patients with BAV compared to controls (5). Our study population was larger and did not show a significant difference in EF compared to controls. Thus, we cannot confirm that there is significant cardiac dysfunction in BAV disease.

All arterial parameters correlated moderately-strongly with age in both groups. While ascending aortic distensibility correlated particularly strong with age in the BAV group, there was no significant correlation for cAIx75 and pAIx75. However, both were abnormal in BAV patients even at young age. This is consistent with earlier findings of impaired ascending aortic elasticity in children with BAV (3). We conclude therefore that proximal arterial stiffness is increased already in childhood and progresses further with age, leading to advanced “arterial age.” PWV and cIMT correlated to the same degree with age in both groups. We therefore propose that these parameters can be used to monitor for cardiovascular risk factors due to arteriosclerosis.

A limitation of this study is that some patients had already undergone aortic surgery including valve replacement. However, prior valve replacement was not associated with any of the parameters analyzed. In addition, the moderate size of our study population in combination with a wide age range may have led to a type II error, especially when performing subgroup-analyses. In the future, we plan longitudinal follow-up of patients who participated in this study.

The clinical impact of this study is that ascending aortic distensibility appears to be the most important predictor of diastolic function in BAV disease. As such, we suggest that clinicians include ascending aortic distensibility in their assessment. Whether pharmacologic amelioration of ascending aortic stiffening and diastolic function is possible should be the subject of future randomized controlled trials.

## Conclusion

Arterial dysfunction in BAV disease is characterized by ascending aortic stiffening, increased wave reflection and central blood pressure. We did not observe general aortic stiffening, cIMT increase or endothelial dysfunction, indicating that arterial stiffening in BAV disease is due to other mechanisms than those seen in acquired heart diseases. Diastolic function appears to correlate best with ascending aortic distensibility in BAV, but overall diastolic function is only marginally decreased. Systolic function is not abnormal either. This argues against an intrinsic myocardial abnormality and for a potentially modifiable interplay between ascending aortic distensibility and diastolic function.

## Data Availability Statement

The raw data supporting the conclusions of this article will be made available by the authors, without undue reservation.

## Ethics Statement

The study was reviewed and approved by Lund University ethics committee. Written informed consent to participate in this study was provided by the particiant and/or the participants' legal guardian.

## Author Contributions

CW: study design, funding acquisition, data acquisition, analysis, and writing and editing manuscript. SL: patient recruitment, data acquisition, and writing and editing of manuscript. AÅ: statistical analyses and review of manuscript. JH: patient recruitment, reviewing, and editing of manuscript. All authors contributed to the article and approved the submitted version.

## Conflict of Interest

The authors declare that the research was conducted in the absence of any commercial or financial relationships that could be construed as a potential conflict of interest.
